# Harnessing Metabolic Reprogramming to Improve Cancer Immunotherapy

**DOI:** 10.3390/ijms221910268

**Published:** 2021-09-24

**Authors:** Liang Yan, Yanlian Tan, Guo Chen, Jun Fan, Jun Zhang

**Affiliations:** 1 Department of Medical Biochemistry and Molecular Biology, School of Medicine, Jinan University, Guangzhou 510632, China; yanl411@stu2020.jnu.edu.cn (L.Y.); tanyanlian@jnu.edu.cn (Y.T.); guochen84@jnu.edu.cn (G.C.); 2 Division of Medical Oncology, Department of Internal Medicine, University of Kansas Medical Center, Kansas City, KS 66160, USA; 3 Department of Cancer Biology, University of Kansas Medical Center, Kansas City, KS 66160, USA

**Keywords:** metabolic reprogramming, tumor microenvironment, tumor immune microenvironment, immune cells, immunosuppression, immunotherapy

## Abstract

Immune escape is one of the hallmarks of cancer. While metabolic reprogramming provides survival advantage to tumor cancer cells, accumulating data also suggest such metabolic rewiring directly affects the activation, differentiation and function of immune cells, particularly in the tumor microenvironment. Understanding how metabolic reprogramming affects both tumor and immune cells, as well as their interplay, is therefore critical to better modulate tumor immune microenvironment in the era of cancer immunotherapy. In this review, we discuss alterations in several essential metabolic pathways in both tumor and key immune cells, provide evidence on their dynamic interaction, and propose innovative strategies to improve cancer immunotherapy via the modulation of metabolic pathways.

## 1. Introduction

Immune cells are an important component of the immune system, which have an important function in the host’s defense against cancer. Immunotherapy has now become a powerful and promising approach to promote the antitumor response [1,2]. However, the function of immune cells can become compromised in the tumor microenvironment (TME), where immune cells face a lack of nutrition, primarily due to competition with tumor cells for a limited nutrient supply [3,4,5]. Furthermore, tumor cells in the TME produce a variety of immunosuppressive metabolites, indicating that metabolic reprogramming plays a critical role in the tumor immune microenvironment (TIME), which determines the response in cancer immunotherapy [6,7,8,9].

Glucose/glycogen metabolism is a major metabolic pathway that is essential for the proliferation, growth and survival of various cells. The rewiring of cell metabolism is an important characteristic of tumor cell development, in which the Warburg effect or aerobic glycolysis is well recognized as a key metabolic hallmark of cancer [5,10,11]. Metabolic alterations in cancer cells provide a selective advantage for survival and proliferation in a unique TME. Furthermore, recent studies have revealed that metabolic reprogramming also occurs in major immune cells within the TME, exhibiting hypoxic and acidotic conditions, including macrophages, T cells and dendritic cells (DCs) [12,13,14,15,16,17]. Tumor-associated macrophages (TAMs) are in general M2-like and facilitate tumor growth through induced immune suppression by enhancing the glycolytic pathway [18,19]. Under a poor glycolytic condition, elevated glutamine and fatty acid consumption supported the M2 polarization of TAMs [20]. T cells are prone to aerobic glycolysis, which is a main metabolic pathway, and aerobic glycolysis is specifically required for the effector function of T cells [21,22,23]. T-regulatory cells (Tregs) and memory CD8^+^ T cells could be induced via lipid oxidation [24,25]. In addition, cellular metabolism also directs the functions of DCs [26,27]. These studies together highlight the importance of metabolic reprogramming in modulating the activity of immune cells.

However, the exact mechanisms underlying the regulation of the antitumor effect of immune cells through metabolic reprogramming remain to be deciphered. In this review, we provide an overview of the metabolic rewiring of immune cells, which relates to their plasticity and corresponding immunomodulatory effect in the tumor microenvironment.

## 2. Metabolic Reprogramming in Cancer Cells

Cellular metabolic reprogramming is critical in the initiation and progression of cancer. A dysregulated vascular system leads to an insufficient supply of key nutrients (such as glucose), resulting in a TME characterized by low glucose, oxygen and pH value. Therefore, in comparison to normal cells, tumor cells tend to shift their metabolic dependency from mitochondrial oxidative phosphorylation to glycolysis, so as to create a favorable environment for survival that provides adequate energy and raw materials for cell division, growth and adaptation induced by oxidative stress—collectively known as the “Warburg effect” [28]. Even though the conversion of glucose into lactate yields only a small amount for ATP, this process can still meet the increased demand for ATP by augmenting glycolysis several folds to compensate for its inefficient extraction of energy from glucose [29]. One theory to explain the Warburg effect is that fast-growing tumors could induce an insufficient oxygen supply, leading to hypoxia and the activation of its downstream of hypoxia-induced transcription factors (HIFs) such as the glucose transporters (GLUT1), phosphoglycerate kinase 1 (PGK1) and vascular endothelial growth factor (VEGF), all necessitating the rewiring of glucose metabolism [30,31,32,33]. Importantly, not only does the TME favor an altered metabolism, the activation of oncogenes and inactivation of tumor suppressor could also drive metabolic changes [34]. For example, KRAS, a well-characterized proto-oncogene when mutated, promotes glycolysis [35,36]. Similarly, the Myc transcription factor can upregulate the expression of various metabolic genes [37], and the p53-mediated regulation of glucose metabolism is dependent on the transcription factor NF-κB [38].

Ample evidence has shown that tumor cells are dependent on metabolic change, e.g., a significant transformation of metabolism from mitochondrial oxidative phosphorylation to aerobic glycolysis [11,39,40]. Such metabolic reprogramming provides survival advantages for cancer cells in the following aspects. First, the rapid entry of glucose into the glycolytic pathway increases the abundance of glycolytic intermediates, enabling them to enter the pentose phosphate pathway (PPP) and provide ribulose for nucleotide synthesis, as well as NADPH, which are critical for redox maintenance and de novo fatty acid synthesis [41]. In addition, limiting the entry of pyruvate into mitochondria is beneficial to the biosynthesis of tricarboxylic acid (TCA) cycle intermediates. This reprogramming may be achieved through the altered activity and/or expression of metabolic enzymes. For example, 6-phosphogluconate dehydrogenase (6PGD) catalyzes the production of ribose-5-phosphate (Ru-5-P), with the latter regulating fatty acid synthesis through inhibiting adenosine monophosphate-activated protein kinase (AMPK) activation via destroying the liver kinase B1 (LKB1) complex [42]. Acetyl-CoA acetyltransferase 1 (ACAT1), a lysine-acetyltransferase, once activated, can inhibit pyruvate dehydrogenase complex (PDC) activity via the acetylation of pyruvate dehydrogenase α1 (PDHA1) and PDH phosphatase 1 (PDP1), thereby inhibiting pyruvate from entering TCA and promoting the survival of tumor cells [43]. Another study also showed that the inhibition of lactate dehydrogenase A (LDHA) prevented the Warburg effect and forced cancer cells to revert to oxidative phosphorylation in order to re-oxidize NADH and produce ATP [44,45]. Although the cells became more respiratorily competent, they exhibited attenuated growth, suggesting that aerobic glycolysis is essential for cancer maintenance and progression [44].

**Figure 1:**
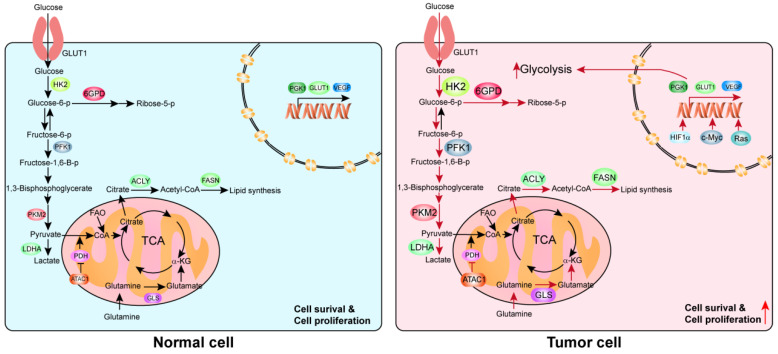
An overview of metabolic reprogramming in normal vs. tumor cells. This diagram depicts the major metabolic pathways in the cell. The red arrows indicate enhanced metabolic processes in cancer cells, whereas the black arrows indicate baseline levels observed in normal cells.

Other than glucose metabolic rewiring, accumulating evidence has also revealed the existence of reprogramming in fatty acid metabolism [46,47] and glutamine metabolism [48,49], and many other metabolic pathways [50,51] in cancer progression (Figure 1).

## 3. Metabolism Reprogramming in Immune Cells

Immune cells play a key role in host defense against infection and cancer. A growing body of evidence also illustrates the importance of cellular metabolism and reprogramming in the proliferation, differentiation and specific function of various types of immune cells.

### 3.1. Macrophages

Macrophages are immune cells with a phagocytic function and are widely distributed in various tissues of the body [52]. They can act as antigen-presenting cells that integrate adaptive and innate immunities [53]. Macrophages display high plasticity, which allows them to switch their phenotype in response to different environmental stimuli [54]. Upon stimulation by IFN-γ or lipopolysaccharide (LPS), macrophages are polarized in the M1 phenotype, whereas M2 polarization can be achieved via incubation with IL-4 and IL-13 [55,56,57]. In TME, macrophages are generally M2, therefore promoting tumor growth by inducing immune suppression [18]. Ample evidence suggests that macrophages display distinct phenotypes during the different stages of tumor development. In the early stage of tumorigenesis, macrophages promote inflammation, whereas they are immunosuppressive in the later stage when the tumor progresses, suggesting the high plasticity of macrophages. It is worth noting that such plasticity makes TAMs an attractive target in cancer immunotherapy.

Current studies suggest that metabolic reprogramming contributes directly to the polarization of macrophages. For example, knockdown of pyruvate dehydrogenase kinase 1 (PDK1) led to diminished M1 macrophages, in which aerobic glycolysis was downregulated, but M2 population of macrophages increased [58]. Pyruvate kinase M2 (PKM2) was activated by promoting tetramers to form to reduce M1 macrophages while increasing the ratio of M2 macrophages [59]. Other studies have also reported that the rate of the TCA cycle and oxidative phosphorylation (OXPHOS) was higher in M2 compared to M1 macrophages.

**Figure 2:**
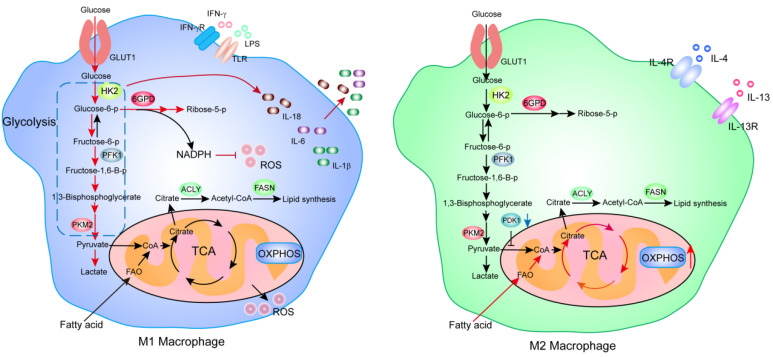
An overview of the difference in cellular metabolic flux between M1 and M2 macrophages. The red arrows indicate enhanced metabolic processes/steps, the blue arrows indicate downregulated processes, and the black arrows indicate baseline metabolic activities.

Rewiring of metabolism is also required to maintain the pro-inflammatory and phagocytic function of macrophages. The LPS-induced M1 macrophage leverages the increased aerobic glycolysis to redirect the carbon flux into the oxidative pentose phosphate pathway (PPP) to produce NADPH—for the purpose of enhancing the phagocytic activity via the generation of reactive oxygen species (ROS) in M1 macrophages. At the same time, glycolysis facilitates the production of cytokines (e.g., IL-6) in M2 macrophages, contributing to pro-inflammatory function [60]. The TCA cycle is broken in M1 macrophages, maintaining the anti-pathogen functions of M1 is ensured by modulating ROS production. In addition, fatty acid oxidation (FAO) is considered to be a key metabolic process in activating the inflammasome and a key signaling event in pro-inflammatory macrophages. The inhibition of FAO suppressed NLRP3 inflammasome activation and the consequent secretion of IL-1β and IL-18 in macrophages [61] (Figure 2).

### 3.2. T Cells

T cells are arguably the most important immune cells, which play an indispensable role in the immune response, mainly through mediating cellular immunity and regulating the immune response of the host [62,63]. There are two main forms of T-cell-mediated cellular immunity: (1) specifically binding and directly killing through destroying the cell membrane of target cells; and (2) releasing cytokines to amplify and enhance the immune effect. T cells are divided into two main subsets based on surface markers and differentiated antigens, including CD4 and CD8 T cells. CD4 T cells are subdivided into T-helper 1 (Th1), T-helper 2 (Th2), T-helper 17 (Th17) and CD4 Treg cells, according to their secreted cytokines and mediated functions [64,65,66,67].

**Figure 3:**
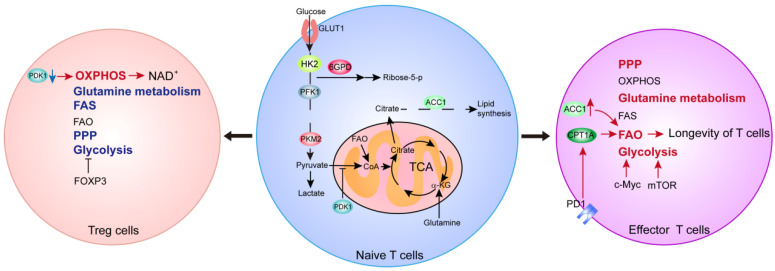
Overview of cellular metabolic pathways in various T cell populations. The red arrows and text indicate enhanced metabolic processes, the blue arrows and text indicate suppressed processes, and the black arrows and text indicate baseline activities.

Metabolic reprogramming is also involved in the survival, proliferation and execution of specific functions of T cells. Firstly, glucose and glutamine metabolism promote T cell activation and functional specialization. Acetylated Foxp3 could enhance oxidative phosphorylation and NAD regeneration by mediating the transcriptional inhibition of Myc to allow Tregs to adapt to low glucose or/and lactate-rich environments [68]. Treg cell stability/function and metabolic homeostasis were regulated by mTORC and c-Myc signaling [69]. The inhibition of PDK1 could decrease Th17 cells and increase Treg populations by enhancing the oxidative phosphorylation of mitochondria, thereby modulating immunity and protecting animals against experimental autoimmune encephalomyelitis [70]. Other studies also showed that the enzyme glucokinase (GCK)-dependent glycolysis regulates Treg cell migration [71]. Furthermore, intracellular enzymes for glucose and glutamine metabolism are upregulated upon T cell activation [72,73]. Glutamine metabolism and intermediates induce the proliferation and differentiation of Th1 cells, Th17 cells and effector CD8^+^ T cells [74,75,76]. The mitochondrial one-carbon metabolism pathway is upregulated upon T cell activation. In fact, serine metabolism could promote T cell proliferation and survival via the flux into one-carbon metabolism [77]. In addition, the regulation of fatty acid synthesis and oxidation is important to T cells. On PD-1 ligation, activated T cells are unable to engage in glycolysis or amino acid metabolism but have an increased rate of FAO, which provides a mechanistic explanation for the longevity of T cells [78]. Finally, acetyl-CoA carboxylase 1 (ACC1) also promotes activation-induced metabolic reprogramming in T cells, as well as Th1 cell and Th17 cell differentiation [79,80]. Altogether, these studies demonstrated that metabolic reprogramming confers the plasticity, and promotes the growth, division and differentiation of T cells (Figure 3).

### 3.3. Dendritic Cells

Dendritic cells (DCs) are professional antigen-presenting cells which provide a bridge from innate to adaptive immunity and initiate the adaptive immune response [81]. Although small in amount, they are widely distributed in various organs of the body other than the brain. Matured DCs highly express major histocompatibility complex I (MHC-I) and MHC-II, and activate CD8 T cells through antigen processing and presentation to mediate antigen-specific cytotoxic effects and improve immune surveillance [82]. Paradoxically, DCs also play a role in inducing and maintaining immune tolerance [83]. Due to the plasticity of DC functions, the investigation of DCs provides potential novel strategies to manage tumor and autoimmune diseases [84,85,86,87,88,89].

**Figure 4:**
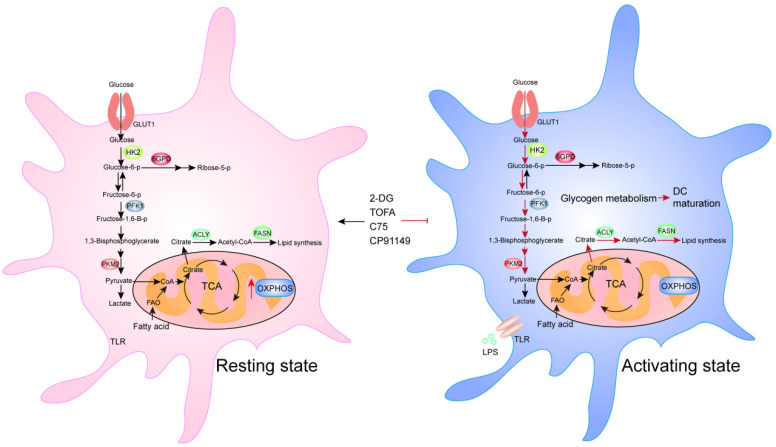
Overview of cellular metabolic plasticity in DCs. The red arrows indicate enhanced metabolic pathways, whereas the black arrows indicate baseline activities.

Compared to resting DCs, activated DCs utilize the increased level of glycolysis to produce more “building blocks” to provide sufficient ingredients for protein and membrane synthesis during DC maturation [15]. Studies have shown that DCs utilize oxidative phosphorylation in their resting state, but shift to glycolysis upon activation [90]. In fact, glycolytic rate rapidly increases after Toll-like receptor (TLR) stimulation to induce DC activation and subsequent survival [91]. The disruption of glycogen metabolism by the glycogen phosphorylase inhibitor CP91149 significantly impairs DC maturation and function. These findings indicate that glycolysis is a key metabolic regulator of DC activation. Treatment with 2-deoxyglucose, a hexokinase (HK) inhibitor, impairs the expression of co-stimulatory markers and the production of IL-12 by conventional DCs (cDCs), which suggested that glycolysis also regulates the function of DCs [92]. Lipid metabolism is also important for DCs. C75 (a fatty acid synthase (FASN) inhibitor) or TOFA (an ACC1 inhibitor) could inhibit DC activation upon LPS stimulation, and reduced the expression of TNF-α and IL-6, which lead to functional impairments, and inactivation of antigen-restricted CD4 T cells or NK cells [93] (Figure 4).

Altogether, these studies demonstrate that metabolic reprogramming confers plasticity in immune cells. This suggests that TME could possibly modulate the metabolic reprogramming of immune cells and reinforce an immunosuppressive microenvironment.

## 4. Relationship between the Metabolism of Cancer and Immune Cells

Immune escape is a hallmark of cancer. Since the TIME is a result of the interaction between cancer and immune cells, it is therefore imperative to understand how metabolites from tumor cells could regulate immune cells, and how metabolic reprogramming in immune cells could modulate their pro- or anti-tumor properties.

### 4.1. Cancer Metabolites Could Inhibit the Functioning of Immune Cells

The success of tumor immunotherapy confirmed the critical role of the host immune system in the antitumor response. However, its therapeutic effect is limited by immune escape of tumor cells. It has been reported that the activation and effector function of immune cells can be regulated by metabolites from tumor cells through various mechanisms.

Firstly, immune cells and tumor cells share common metabolic pathways. Tumor cells therefore could affect immune cell function by competitively consuming nutrients in the TME. For example, cancer cells can metabolically restrict T cells by competitively consuming glucose, leading to the inhibition of the glycolytic capacity and IFN-γ production in T cells, which permits tumor progression. Interestingly, immune checkpoint blockade against CTLA-4, PD-1 or PD-L1 was found to restore glucose availability in the TME, and to permit T cell glycolysis and IFN-γ production [5]. Consistently with this, glucose starvation also reduces IFN-γ production by tumor-infiltrating CD8^+^ T cells [22]. In addition, glutamine, tryptophan and arginine can also provide nutrition for immune cells, and a deficiency of these amino acids can lead to an immunosuppressive TME through various mechanisms [1]. Altogether, these findings suggest that immune escape can be induced when the nutrition supply to immune cells is competitively consumed by cancer cells.

In addition, tumor cells produce a variety of metabolites leading to the dysfunction of immune cells in the TME. As cancer cells tend to undergo aerobic glycolysis, a large amount of lactate is produced and secreted. Tumor-derived lactate can inhibit the movement, cytotoxicity and effector function of T cells, and a reducing level of LDHA-associated lactic acid was found to be an effective way to restore T cell function [94,95]. A high lactate concentration results in a low-pH microenvironment that is detrimental to the survival, differentiation and migration of immune cells, therefore interfering with their function [8,96,97]. Tumor-derived lactate could also promote the apoptosis of naive T-cells through suppressing the expression of FAK-family-interacting protein of 200 kDa (FIP200), leading to the evasion of immunity [98]. In addition, breast cancer tissue-derived free fatty acids could inhibit cytotoxic T-lymphocyte-mediated killing to reduce antitumor activity [99], and some tumor-derived amino acids (such as tryptophan and arginine), nucleotides (such as adenosine) and intermediates of TCA (such as α-ketoglutarate) could also have an inhibitory effect on the activation or function of immune cells, promoting the evasion of immunity [2,100]. Furthermore, tumor cells could disrupt methionine metabolism in CD8^+^ T cells, leading to lower intracellular levels of methionine and the methyl donor S-adenosylmethionine (SAM), as well as the loss of H3K79me2, resulting in the low expression of STAT5 and impaired T cell immunity [101].

TME could repress T cell mitochondrial biogenesis to drive metabolic insufficiency and dysfunction of intratumoral T cells [102]. In addition, tumor cells were found to be capable of converting naive/effector T cells into senescent T cells to induce immune tolerance, a process that is dependent on tumor-derived endogenous metabolic cAMP [103]. Importantly, other immune cells can be affected as well. For example, a recent study suggested that hepatocellular carcinoma (HCC)-derived ectosomal PKM2 cells promote metabolic reprogramming in monocytes and nuclear STAT3 phosphorylation to upregulate specific transcription factors, leading to monocyte-to-macrophage differentiation and tumor microenvironment remodeling [104]. In other studies, the inhibition of tumor glutamine metabolism could decrease the recruitment and infiltration of MDSCs by increasing cell death and decreasing tumor CSF3 expression, and this led to an increase in inflammatory TAM differentiation and inhibited tumor growth [105].

### 4.2. Metabolic Reprogramming of Immune Cells Affects Tumor Progression

The tumor microenvironment is challenging to the survival and function of immune cells, and necessitates their metabolic adaptation. Therefore, metabolic alterations in immune cells could also impact tumor progression.

TAMs are closely related to the initiation and development of tumors. Some studies have demonstrated that TAMs exhibit an increase in aerobic glycolysis, with an inflammatory phenotype and pro-tumorigenic effects. Hexokinase 2 (HK2) inhibitor 2-DG was sufficient to disrupt these effects [106]. PFKFB3, a key glycolytic enzyme, is upregulated in tumor-associated monocytes, and leads to the activation of glycolysis, and this can induce PD-L1 expression, and subsequently attenuate the response of cytotoxic T lymphocyte in tumor tissues [107]. In macrophages, the CpG oligodeoxy-nucleotide, an agonist of Toll-like receptor 9, could promote de novo lipid biosynthesis that enables antitumor activity, including the engulfment of CD47^+^ cancer cells [108]. Another study also showed that a deficiency of receptor-interacting protein kinase 3 (RIPK3) in TAMs facilitated FAO and induced M2 polarization in the tumor microenvironment. On the contrary, the upregulation of RIPK3 inhibited FAO via the suppression of PPAR activation, and could reverse the immunosuppressive activity of TAMs and dampen HCC tumorigenesis [109], and inhibition of FAO in TAMs promoted the anti-tumorigenic differentiation of TAMs and inhibited tumor growth [110]. These findings demonstrate that the metabolic reprogramming of TAMs plays a role in regulating tumor progression.

T cells in the TME. It is well known that a glucose-deficient tumor microenvironment limits aerobic glycolysis in tumor-infiltrating T cells, which suppresses tumoricidal effector functions [111]. For this purpose, tumor-specific CD4 and CD8 T cells were shown to increase their effector functions to slow tumor growth by increasing PEP production through the over-expression of phosphoenolpyruvate carboxykinase 1 (PCK1) [111]. Other evidence suggests that Treg cells, which mainly rely on FAO rather than glycolysis, could survive under these conditions and exert their immunosuppressive effect [24]. Endogenous fatty acid synthesis and the glycolytic–lipogenic axis are important for Th17 cell development [79]. Enhancing CD8^+^ T cell fatty acid catabolism could preserve effector functions of CD8^+^ TILs when subjected to hypoglycemia and hypoxia, thereby maintaining the efficacy of melanoma immunotherapy [112]. Finally, memory CD8+ T cells could more efficiently slow tumor progression through the preference of FAO and OXPHOS for energy production [113,114].

Antitumor immune response also depends on the adequate function of host DCs which could be regulated by metabolic alterations. The lipid accumulation in DCs dampens their ability to process and present tumor antigens, but treatment with 5-(tetradecycloxy)-2-furoic acid (TOFA, an inhibitor of acetyl-CoA carboxylase) could restore the activity of DCs to effectively stimulate T cells and enhance anti-cancer effect [26]. In addition, activation of XBP1 could induce a triglyceride biosynthetic program leading to abnormal lipid accumulation in tumor-associated DCs (tDCs), and subsequently inhibiting tDC capacity, thereby driving ovarian cancer progression [115].

**Figure 5:**
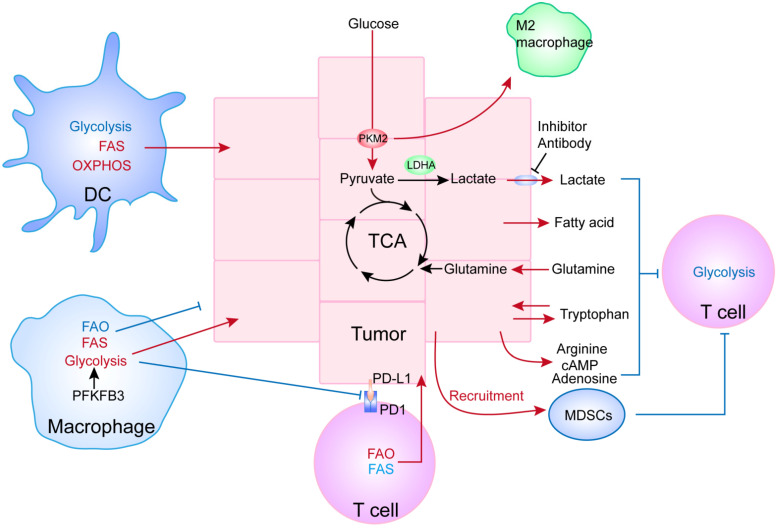
Crosstalk and interplay between tumor and immune cells through metabolic reprogramming. This illustration shows that the reprogramming of certain metabolic pathways in tumor cells, such as glycolysis, can offer not only a survival advantage to the tumor cells, but can also change the behavior/function of immune cells. Similarly, the metabolic rewiring of immune cells can act on tumor cells reciprocally to facilitate tumor progression. The red arrows and text indicate enhanced metabolic processes, whereas the blue arrows and text indicate suppressed activities. The black arrows and text indicate baseline levels.

Taken together, it is obvious that TME modulates the metabolic reprogramming of immune cells, which in turn could have various impacts on tumor growth. Such observations could therefore provide innovative strategies to reduce tumor immune-tolerance, which is fostered by an immune-suppressive TME (Figure 5).

## 5. The Metabolic Crosstalk between Cancer-Associated Immune Cells and Cancer Stem Cells

The tumor-regenerating and tumor-propagating activities are in general associated with cancer stem cells (CSCs), or stem-like cells, the minor population of cells that are responsible for immune evasion, therapeutic resistance and disease recurrence. Recently, the immune evasion of CSCs has been linked to the change of metabolism/metabolites [116].

Such metabolic reprogramming is believed to confer the plasticity in CSCs [117]. For example, voltage-dependent anion channel 2 (VDAC2) regulates glucose metabolism and reprogramming by interacting with PFKP to impair self-renewal and tumorigenic properties of glioma stem cells [118]. Oxidized ATM promotes breast cancer stem cell enrichment through facilitating the glycolytic flux to mitochondrial pyruvate and citrate and regulating ATP-citrate lyase (ACLY) activity, resulting in acetyl-CoA accumulation in the cytoplasm [119]. HectH9 deficiency controls CSC expansion by inhibiting the K63-linked ubiquitination of HK2 to impede tumor glucose metabolism [120].

Interestingly, tumor-associated immune cells are closely related to the number, drug resistance and tumorigenicity of CSCs [121]. For example, metabolic reprogramming was found to directly affect macrophages’ polarization to the M2 phenotype [58,59], and glycolysis facilitates the secretion of IL-6 in M2 macrophages [60], IL-6 then enriches cancer cells with the CSC phenotype [122]. Several recent observations showed that tumorigenic DCs provided pro-survival signals to maintain CSCs in solid and hematological malignancies by targeting neurospheres [123]. In turn, CSCs can induce M2 phenotype in TAMs, subsequently suppressing anti-tumor CD8+ responses to increase chemotherapeutic resistance [124]. Deletion of Arf1 in mice was found to disrupt lipid metabolism resulting in lipid droplet accumulation, which selectively eliminated CSCs, activated DCs via increased release of damage-associated molecular patterns (DAMPs), and further enhanced T-cell infiltration and activation to stimulate an anti-tumor immunity [125].

## 6. Potential Novel Strategies for Cancer Immunotherapy

**Figure 6:**
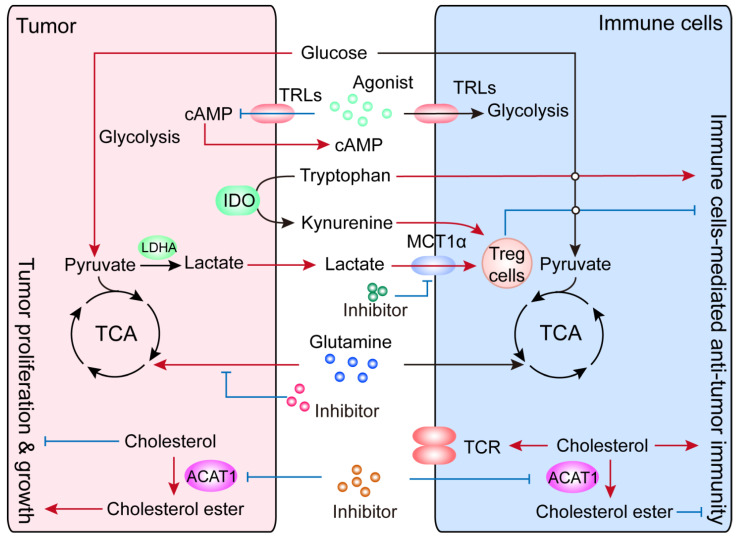
An overview of potentially innovative strategies to improve cancer immunotherapy through the manipulation of metabolic reprogramming. Glutamine, ATAC1, IDO, lactate and TLRs might be considered novel “metabolic checkpoints”, the targeting of which could potentially enhance the function of immune cells to better achieve anti-tumor effects. The red arrows indicate enhanced metabolic processes or functions, the blue arrows indicate suppressed processes, and the black arrows and text indicate baseline activities.

As mentioned above, cellular metabolism is not only important for the proliferation and survival of cancer cells (including CSCs), but is also crucial for the differentiation, survival and function of immune cells. As tumor cells constantly adjust their metabolism and nutrient acquisition to sustain maintenance/progression and compete for nutrients with immune cells, there is a constant need for metabolic rewiring in tumor-infiltrating immune cells that could potentially result in immune-suppression and immune escape. Harnessing metabolism could therefore provide innovative approaches to improve cancer immunotherapy (Figure 6).

Glutamine, a nonessential amino acid, is the most abundant nutrient in the blood. Cancer cells require high concentrations of glutamine, which is necessary for supporting robust cell proliferation—in fact, cancer cells are more dependent on glutamine [126]. In addition, glutamine is also an essential substrate and provides pro-survival signaling for the activation and growth of T cells [127]. Glutamine catabolism could supply the intermediate metabolites and substrates required for cells, and it is intensely induced in active T cells to regulate the effects of T cells [128]. It has been reported that glutamine deprivation resulted in the activation of naïve CD4^+^ T cells, differentiating into Tregs, and the addition of α-KG reversed this effect and increased the rate of Th1 differentiation under conditions of glutamine deprivation [76]. A recent study showed that by applying a small-molecule inhibitor of glutamine metabolism, researchers were able to not only inhibit tumor growth, but also markedly suppress the generation and recruitment of MDSCs [105]. Others showed that the compound JHU083 blocked glutamine metabolism in tumor-bearing mice to suppress the oxidative and glycolytic metabolism of cancer cells; In contrast, a markedly up-regulated oxidative metabolism, and highly activated phenotype were observed in effector T cells [129]. Currently, the glutaminase inhibitor telaglenastat (CB-839) is being investigated in various cancer clinical trials with or without the combination of immunotherapy [130].

Toll-like receptors (TLRs), an evolutionarily ancient family of pattern recognition receptors, are one of the major pattern recognition receptors (PRRs) expressed by immune cells, as well as non-immune cells, including cancer cells [131]. TLRs are abundantly expressed in these cells. More recent studies suggest that TLRs may directly regulate cell metabolism, therefore affecting tumor behaviors in TME. For example, TLR3 stimulation could induce OXPHOS to be converted into glycolysis in tumor cells, supporting the tumor’s adaptation to hypoxia [132]. TLR9 has been suggested to regulate lipid peroxidation in response to oxidative stress in patients with breast cancer [133]. Furthermore, TLR-mediated rewiring of cell metabolism is critical for the activation and function of immune cells. LPS stimulation could promote the switch from OXPHOS to glycolysis in macrophages via TLR4 [134]. Similarly, TLRs could increase glycolysis and reduce OXPHOS in DCs [134]. Other studies have also described that activated TLR1 and TLR2 could increase the glycolysis and proliferation of Tregs [135]. It is worth noting that TLRs can also indirectly influence anti-tumor immune responses in the TME. A study showed that activated TLR8 could prevent cAMP production in tumor cells and block the tumor-induced conversion of naive and tumor-specific T cells into senescent cells, therefore enhancing antitumor immunity in vivo [136]. Altogether, these studies indicate that TLRs are critical to mediate the function of immune cell and cancer cells in TME.

Acetyl-CoA acetyltransferase 1 (ACAT1) is a key cholesterol esterification enzyme that converts free cholesterol into cholesterol ester. It has been reported that a small-molecule ACAT1-specific inhibitor could reduce the inflammatory response with the induction of LPS [137]. The modulation of membrane lipids can also affect T cell activity [138]. Knocking out or inhibiting of ACAT1 could enhance the immune response of CD8^+^ T cells by increasing TCR clustering and signaling by upregulating the level of cholesterol in the membrane. Consistently with this, the ACAT inhibitor avasimibe was used to treat melanoma in mice, and it could effectively inhibit tumor progression [139]. Similarly, down-regulating ACAT1 expression or avasimibe administration could efficiently inhibit the growth and metastasis of Lewis lung carcinoma via potentiating the anti-tumor response of CD8^+^ T cells [140]. In triple-negative breast cancer, the inhibition of ACAT1 expression could reduce tumor growth in TNBC mammospheres [141]. Finally, ACAT1 inhibitor or shRNA knockdown significantly suppressed tumor growth and metastasis in an orthotopic mouse model of pancreatic cancer [142]. All these studies indicate that ACAT1 could serve as a potential target to improve cancer immunotherapy in the future.

Generally, lactate could be produced through glycolysis in low-oxygen conditions and is considered to be a futile product for cancer cells’ proliferation and survival. However, several studies have broadened the metabolic function of lactate to include its contribution to the TCA cycle [143,144,145], the migration and invasion of cancer cells [146] and the activation of multiple oncogenic signaling pathways [147]. Furthermore, lactate has been shown to acidify the TME and compromise the functions of various immune cells, leading to escape from immune-surveillance [95,148,149]. Consistently with these observations, a recent study showed that Treg cells can adapt to high-lactic-acid conditions and use lactic acid to produce intermediate products to provide conditions for proliferation. In fact, lactate uptake was found to be indispensable for Tregs, and deficiency or inhibition of MCT1-α lactate transporter in Treg cells induced tumor growth retardation and enhanced immune response [150]. In addition, released of lactate by glycolytic cancer-associated fibroblasts (CAFs) can reduce the percentage of the antitumoral Th1 subset and increase Treg cells via NF-κB activation and FoxP3 expression in prostate cancer [151]. Lactate secretion can also be induced by PKM2 up-regulation, resulting in increased expression of Galectin-9 through NF-κB signaling to promote tumor progression—knocking down Galectin-9 could notably enhance the killing effect of NK cells [152].

Indoleamine 2,3-dioxygenase (IDO) mediates the conversion of tryptophan to kynurenine in tumor cells. Over-expression of IDO can inhibit the antitumor immune response of T cells by increasing the kynurenine to tryptophan ratio in TME. Blocking IDO can therefore reduce Treg cells and restore the function of T cells [153,154]. Thus, IDO inhibitors are used as a new type of immunomodulatory drug to activate T cells, and improve immune response via its impact on tryptophan metabolism. Currently, several clinical trials are ongoing to investigate the therapeutic value of combining IDO inhibitors with immunotherapy or chemotherapy, including epacadostat, indoximod, navoximod, EOS200271 and BMS-986205, were used to combine with anti-PD-1/CTLA4 anti-bodies or chemotherapy in clinical trials, testing their efficiency in patients [155,156]. Regrettably, epacadostat combined with anti-PD-1 antibody was announced to lack the efficiency for melanoma treatment [157], however it was found to be safe in combination with immune checkpoint inhibitor pembrolizumab, and its value needs to be further explored in the context of identifiable biomarkers and tumor types.

## 7. Conclusions

While cancer cells are constantly reprogramming major metabolic pathways (e.g., glycolysis, PPP, fatty acid oxidation and glutamine metabolism, etc.), such metabolic rewiring also occurs in tumor-infiltrating immune cells to modulate their pro- or anti- tumor functions. Through above-mentioned discussion, we have demonstrated that cellular metabolism confers plasticity in both the immune and cancer cells, and there exists mutual metabolic regulation for which we can potentially harness to improve cancer immunotherapy through innovative strategies. Although studies suggest that glutamine, ATAC1, lactate, TLRs and IDO could be exploited as “metabolic checkpoints” for therapeutics, when and how to combine them with current standard-of-care immunotherapy to achieve optimal value needs further investigation. Nevertheless, we are optimistic that a deep understanding of the metabolic mechanisms underlying immune evasion will help provide a blueprint that aims to preserve immune cell function while simultaneously inhibiting cancer cells via modulation of essential metabolic pathways.

## Data Availability

All figures in this manuscript are non-published and original.
